# A genome-wide association study of coat color in Chinese Rex rabbits

**DOI:** 10.3389/fvets.2023.1184764

**Published:** 2023-08-16

**Authors:** Kai Zhang, Guozhi Wang, Lihuan Wang, Bin Wen, Xiangchao Fu, Ning Liu, Zhiju Yu, Wensu Jian, Xiaolin Guo, Hanzhong Liu, Shi-Yi Chen

**Affiliations:** ^1^Sichuan Academy of Grassland Sciences, Chengdu, Sichuan, China; ^2^Farm Animal Genetic Resources Exploration and Innovation Key Laboratory of Sichuan Province, Sichuan Agricultural University, Chengdu, Sichuan, China

**Keywords:** Rex rabbits, coat color, GWAS, RAD-seq, genome

## Abstract

Coat color is an important phenotypic characteristic of the domestic rabbit (*Oryctolagus cuniculus*) and has specific economic importance in the Rex rabbit industry. Coat color varies considerably among different populations of rabbits, and several causal genes for this variation have been thoroughly studied. Nevertheless, the candidate genes affecting coat color variation in Chinese Rex rabbits remained to be investigated. In this study, we collected blood samples from 250 Chinese Rex rabbits with six different coat colors. We performed genome sequencing using a restriction site-associated DNA sequencing approach. A total of 91,546 single nucleotide polymorphisms (SNPs), evenly distributed among 21 autosomes, were identified. Genome-wide association studies (GWAS) were performed using a mixed linear model, in which the individual polygenic effect was fitted as a random effect. We detected a total of 24 significant SNPs that were located within a genomic region on chromosome 4 (OCU4). After re-fitting the most significant SNP (OCU4:13,434,448, *p* = 1.31e-12) as a covariate, another near-significant SNP (OCU4:11,344,946, *p* = 7.03e-07) was still present. Hence, we conclude that the 2.1-Mb genomic region located between these two significant SNPs is significantly associated with coat color in Chinese Rex rabbits. The well-studied coat-color-associated agouti signaling protein (*ASIP*) gene is located within this region. Furthermore, low genetic differentiation was also observed among the six coat color varieties. In conclusion, our results confirmed that *ASIP* is a putative causal gene affecting coat color variation in Chinese Rex rabbits.

## Introduction

Among all farm animals, modern rabbits (*Oryctolagus cuniculus*) are among the most recently domesticated species, although the exact domestication date of the species remains controversial when examined on the basis of archeological records and genetic evidence (1, 2). However, it has been widely acknowledged that modern rabbits have a single domestication origin, resulting in lower genetic variation in comparison with other farm animals (3–5). More than 200 rabbit breeds have been officially registered in the Domestic Animal Diversity Information System (DAD-IS),^1^ and these show considerable morphological variation, such as in body size, coat color, and hair phenotype (6, 7). Among them, the Rex rabbit is well known for its short, dense, and smooth hair. This Rex rabbit phenotype is believed to have genetically originated from normal hair (8). Coat color is an important phenotypic trait in the fur industry, and at least 16 color varieties of Rex rabbits have been recognized by the American Rabbit Breeders Association (ARBA);^2^ however, the preferred coat color differs between different markets.

Coat color in mammals is determined by the relative amounts of eumelanin and phaeomelanin in melanocytes, and many studies have been conducted to identify coat-color-associated genes and causal mutations in domestic animals during the past two decades (9). In domestic rabbits, melanocortin 1 receptor (*MC1R*) is the first gene to have been thoroughly studied, and several causal mutations have been successfully identified as affecting coat color (10, 11). As a competitive ligand to *MC1R* in the melanin synthesis pathway (12), a premature stop mutation of the agouti signaling protein (*ASIP*) gene has been reported to be responsible for the non-agouti black coat color in rabbits (13). Additionally, a premature stop mutation of the tyrosinase-related protein 1 (*TYRP1*) gene is associated with brown coat color in rabbits (14). Based on the gene expression patterns observed in Rex rabbits of various colors, it has been suggested that the POU class 2 homeobox 1 gene (*POU2F1*) affects fur color formation in Rex rabbits (15). The variability of the tyrosinase (*TYR*) gene has been studied in domestic and wild European rabbits; this work has confirmed the effects of missense mutations on coat colors (16). The genetic polymorphisms of five candidate genes were genotyped to investigate their associations with different coat colors in rabbits (17). In addition to loci determining different coat colors, it was found that both eumelanic and pheomelanic pigmentations can be further diluted more or less under genetical control of the *dilute* locus: for example, black can be diluted to gray. Fontanesi et al. (18) successfully mapped the *dilute* locus of rabbits to the melanophilin (*MLPH*) gene and identified a frameshift mutation associated with the dilute coat color.

Due to the wide application of high-throughput sequencing technologies, large numbers of genome-wide variants can now be discovered and genotyped at an affordable cost (19). Among these technologies, restriction-site-associated DNA sequencing (RAD-seq) is a cost-efficient approach for investigating genome-wide variants, especially in non-model species (20); the approach was first proposed in 2008 and is characterized by sequencing of small genomic fragments that are randomly digested by restriction enzyme (s). The RAD-seq approach has been widely used for population genetics and genome-wide association studies (GWAS) [such as by (21–24)]. In rabbits, genetic diversity and population structure have been investigated using genome-wide single nucleotide polymorphisms (SNPs) that have been generated using the RAD-seq approach (25, 26). In this study, we similarly employed the RAD-seq approach to identify genome-wide SNPs, which we subsequently used for GWAS with six coat color varieties of Chinese Rex rabbits. The results could help us to better understand the underlying genetic basis of coat colors in Chinese Rex rabbits.

## Materials and methods

### Animals and genomic DNA

**Figure 1:**
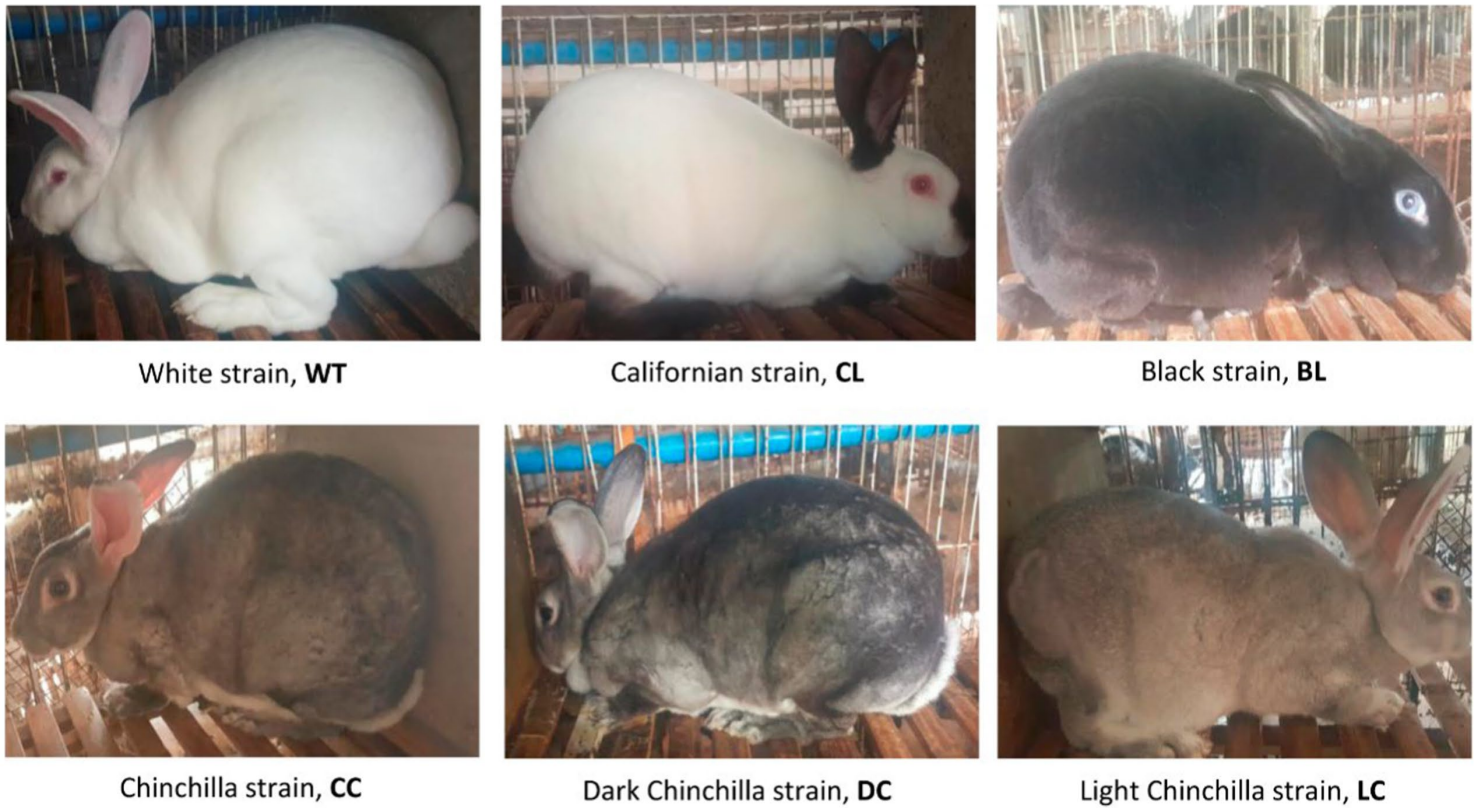
Phenotypes of the six coat color varieties of Rex rabbit included in this study.

Venous blood was collected from the marginal ear veins of 250 Rex rabbits raised at the Research Farm of Sichuan Academy of Grassland Sciences. The rabbits consisted of six coat color varieties: 40 White Rex (WT), 42 Californian Rex (CL), 42 Black Rex (BL), 42 Chinchilla Rex (CC), 42 Dark Chinchilla Rex (DC), and 42 Light Chinchilla Rex (LC). Among these varieties, WT, CL, BL, and CC exhibit different coat colors, and there are two varieties of CC with a darker (DC) and lighter (LC) coat color, respectively (Figure 1). There was no genetic relationship within three generations among any of the sampled animals according to pedigree information. Genomic DNA was extracted using the Axy-Prep Genomic DNA Miniprep Kit (Axygen Bioscience, USA).

### Genome sequencing

Based on preliminary investigation on the reference genome sequences of rabbits, the restriction enzyme *EcoRI* (NEB, Beijing) was successfully used to digest genomic DNA (~1 μg per sample used). Sequencing libraries were constructed according to the recommended pipeline (20). In brief, P1 adapter sequence was first added to the digested fragments; this was followed by sequential steps of sample pooling, random shearing, and fragment size-based selection using agarose gel. Subsequently, DNA was ligated to a second adapter (P2) with divergent ends. DNA fragments of ~400 bp in length were selected to construct the sequencing libraries. Finally, the libraries were sequenced on an Illumina HiSeq platform and 150 bp paired-end reads were generated (Novogene Co. Ltd., Beijing).

### Reads mapping and SNP genotyping

After the initial sequencing images were converted into sequence files in the FASTQ format using a standard pipeline, we first investigated the Q_phred_ value-based error rate. Using the fastp software package (27), low-quality reads were discarded according to three criteria (26): (1) reads containing adaptor sequences, (2) reads containing ambiguous bases for more than 10% of the total length, and (3) reads containing low-quality bases (Q_phred_ value <5) for more than 50% of the total length. If either member of the paired reads was marked as low quality, both pairs were discarded. After these steps, we obtained clean reads and subjected them to the following analyses.

All clean reads were mapped to a rabbit reference genome (UM_NZW_1.0) using the BWA software with default parameters (28). Subsequently, we employed the GATK toolkit v3.8 (29) for discovery and genotyping of small variants (SNPs and InDels) among all samples according to GATK Best Practices recommendations (30, 31); in this process, the duplicate removal, realignment, and hard filtering steps were performed with default parameters. After exclusion of all InDels, a raw set of SNPs was obtained. SNPs were removed if they had a coverage depth < 3, calling rate < 90% for the genotypes or individuals, minor allele frequency (MAF) < 0.05, and extreme deviation from Hardy–Weinberg equilibrium (HWE, *p* < 10^−8^). Finally, we extracted biallelic SNPs and generated a clean set of SNPs. The missing genotypes were further imputed using the Beagle software package v5.4 with default parameters (32).

### Population and association analyses

We investigated the genomic distribution of clean SNPs and the transition/transversion ratio using the ANNOVAR software package (33). Nucleotide diversity (π) for each locus was calculated using the vcftools software package (34). The PopSc toolkit (35) was used to calculate the polymorphism information content (PIC), inter-variety Wright’s F_ST_, and intra-variety Wright’s F_IS_ (36). The pairwise *p*-distances among all samples were calculated from all SNPs using the TreeBeST software package (TreeSoft) and then subjected to the construction of a phylogenetic tree according to the neighbor-joining method (37); this phylogenetic tree was visualized using the ggtree R package (38).

In GWAS, the six coat color varieties of Rex rabbits were arbitrarily coded using the ordinal values of WT = 1, LC = 2, CC = 3, DC = 4, CL = 5, and BL = 6. To avoid potential bias arising from the arbitrary coding of coat colors, the reverse order was employed in independent repeat performance of GWAS. The effect of each SNP was estimated using a mixed linear model implemented in the GCTA software package (39):


y=1+Zβ+Wμ+e

where y is the vector of coat colors coded above; 1 is the mean term ; β is the fixed effect of the SNP tested for association; Z is a vector containing the genotype score for the tested SNP; μ is the vector of individual random polygenic effects with $\mu \sim N\left(0,G\sigma_{u}^{2}\right)$, where G is the genomic relationship matrix and $\sigma_{u}^{2}$ is the additive genetic variance; W is the incidence matrix for μ; and e is a vector of random residual effects with $e\sim N\left(0,I\sigma_{e}^{2}\right)$, where I is an identity matrix and $\sigma_{e}^{2}$ is the residual variance. After estimation of the SNP effects, the most significant SNP was selected and further added as a covariate to the mixed linear model described above. A Bonferroni approach was used for correction of multiple comparisons in the GWAS results (40).

## Results

### Sequencing and SNPs

**Figure 2:**
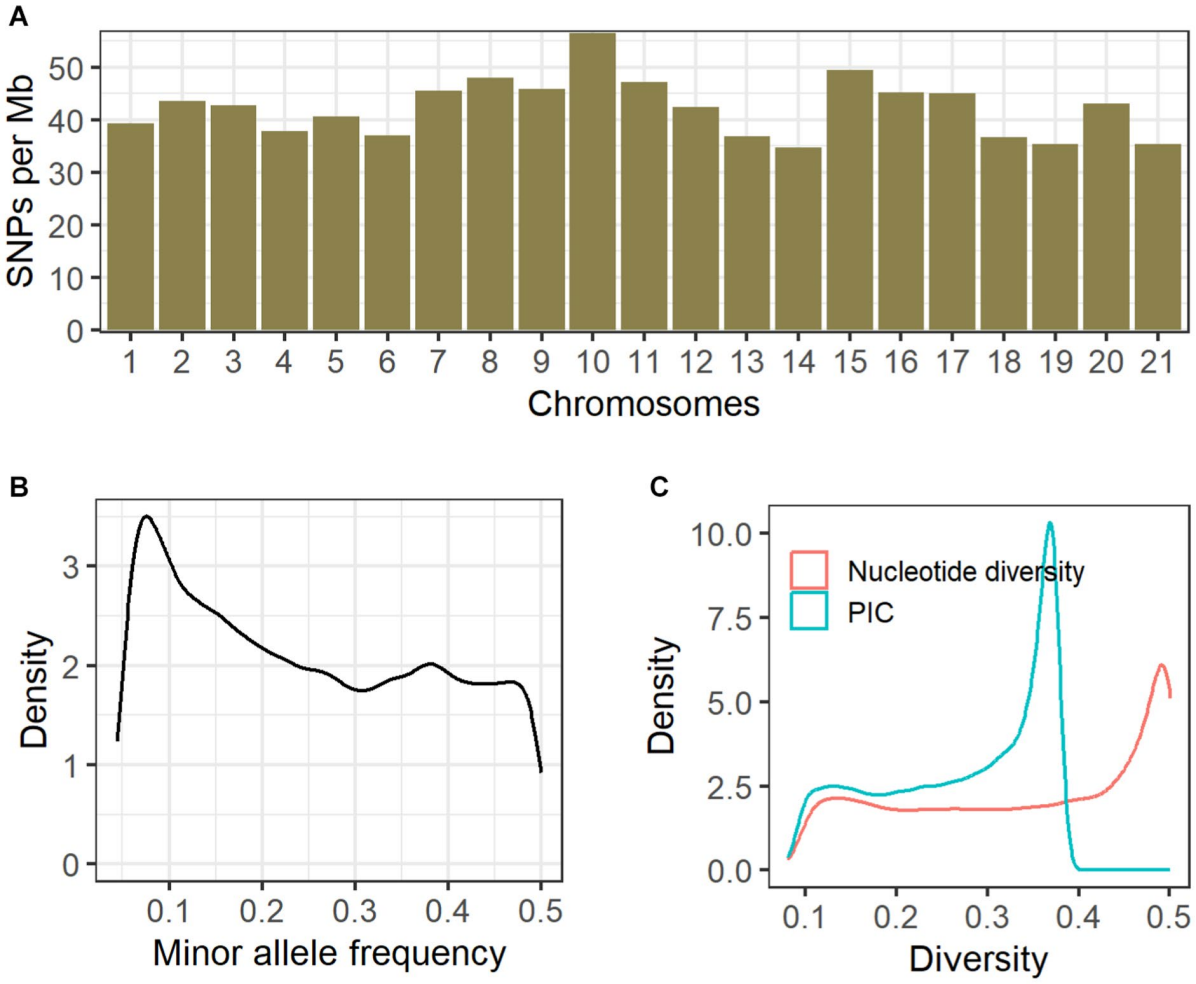
Genomic distribution and genetic diversity of SNPs. For all clean SNPs, we investigated the genomic distribution **(A)**, minor allele frequencies **(B)**, and the density distribution of nucleotide diversity and polymorphism information content **(C)**.

We obtained 208.51 Gb raw paired-end reads (approximately 1.5 billion reads) across all the sequenced samples, from which 208.48 Gb clean reads (0.83 Gb per sample) were generated after the quality control steps. On average, 98.9% of the clean reads were successfully aligned against the reference genome. A total of 5,162,522 raw SNPs were generated on 21 autosomes, and we finally obtained 91,546 high-quality biallelic SNPs according to our custom filtering process. These SNPs were distributed across the whole genome, and an average of 42.3 SNPs per Mb genomic region was comparably observed among all autosomes (Figure 2A). The mean MAF was ~0.25 (Figure 2B). There were 64,311 transitions and 27,235 transversions (transition/transversion ratio = 2.36). Using the reference annotation of the rabbit genome (UM_NZW_1.0), we inferred the locations of the SNPs. SNPs were distributed within exons (*N* = 1,648), introns (*N* = 34,037), 1 kb upstream/downstream regions of genes (*N* = 1,411), and intergenic regions (*N* = 54,450).

### Genetic diversity and population structure

Among all clean SNPs, the distribution density of nucleotide diversity exhibited a single peak close to 0.5, and a similar pattern was also observed for PIC (Figure 2C). The median and mean values for nucleotide diversity across the six coat color varieties were 0.3518 and 0.3185, respectively (Table 1); among these, the Black Rex showed the highest degree of nucleotide diversity, with a median of 0.3672 and a mean of 0.3340. The Black Rex and Californian Rex had the highest and lowest PIC, with mean values of 0.2649 and 0.2440, respectively. Furthermore, there were no obvious differences among the coat color varieties in relation to genetic diversity.

**Table 1:** Nucleotide diversity (π) and polymorphism information content (PIC) in different coat color varieties of Rex rabbit.

Coat color variety	π		PIC	
	Median	Mean	Median	Mean
White Rex	0.3532	0.3236	0.2879	0.2575
Californian Rex	0.3408	0.3069	0.2800	0.2440
Black Rex	0.3672	0.3340	0.2970	0.2649
Chinchilla Rex	0.3543	0.3169	0.2888	0.2520
Dark Chinchilla Rex	0.3408	0.3116	0.2800	0.2478
Light Chinchilla Rex	0.3543	0.3179	0.2888	0.2526
**Overall**	**0.3518**	**0.3185**	**0.2871**	**0.2531**

**Figure 3:**
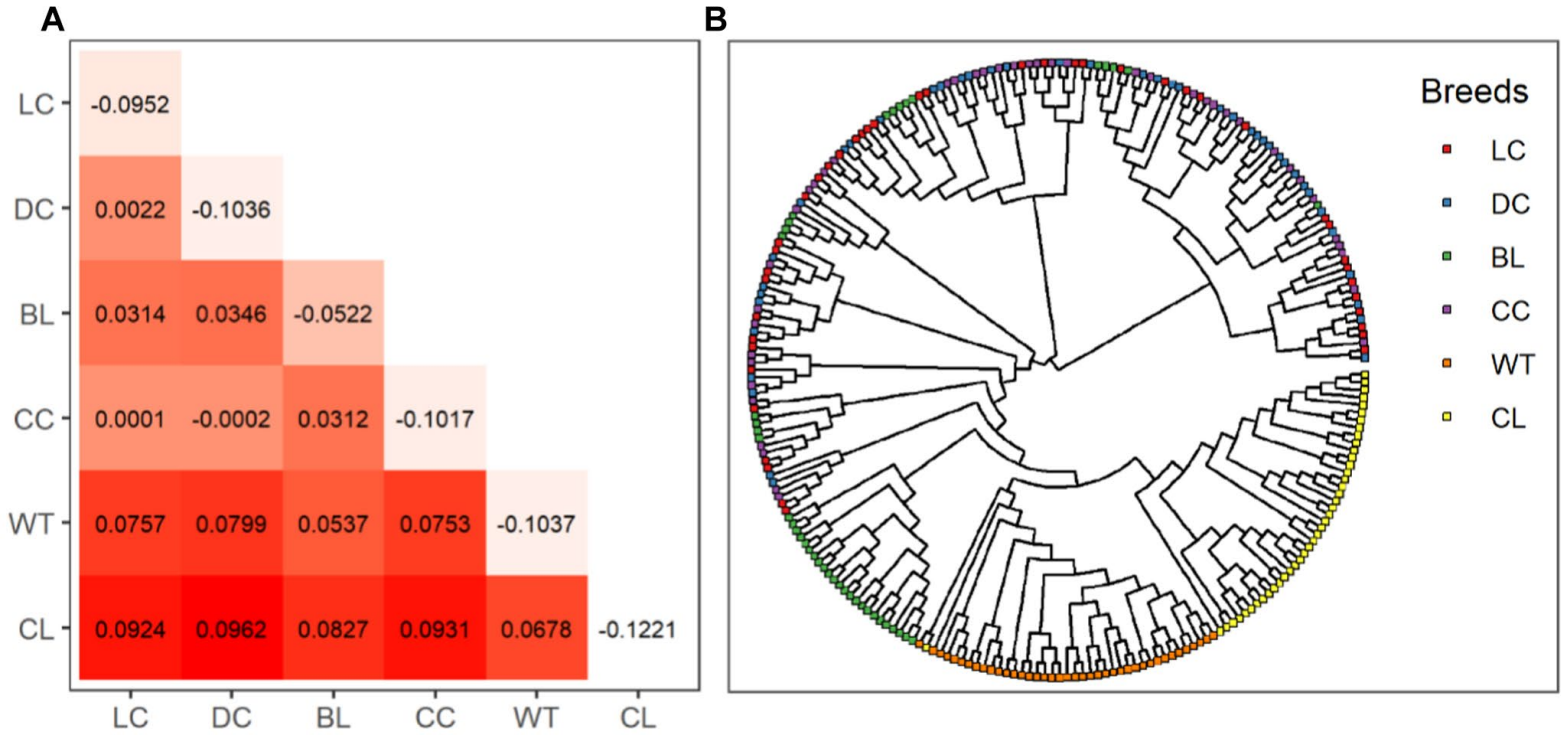
Genetic structures among the six coat color varieties of Rex rabbit. The matrix **(A)** shows pairwise Wright’s F_ST_ values in the lower triangle and F_IS_ values in diagonal cells. The phylogenetic tree for all 250 animals is shown in **(B)**. WT, White Rex; CL, Californian Rex; BL, Black Rex; CC, Chinchilla Rex; DC, Dark Chinchilla Rex; LC, Light Chinchilla Rex.

The highest and lowest degrees of inter-variety differentiation were observed between the Dark Chinchilla Rex and the Californian Rex (F_ST_ = 0.0962), and between the Chinchilla Rex and the Dark Chinchilla Rex (F_ST_ = −0.0002), respectively (Figure 3A). Intra-variety inbreeding coefficients (F_IS_) ranged from −0.1221 in the Californian Rex to −0.0522 in the Black Rex. According to the phylogenetic tree for all samples (Figure 3B), both the White Rex and the Californian Rex formed their own clusters and were separated from the other breeds. Next, most of the Black Rex rabbits were clustered together and were almost distinguishable. However, there was no obvious clustering pattern among the Chinchilla Rex and the other two breeds.

### Association with coat colors

**Figure 4:**
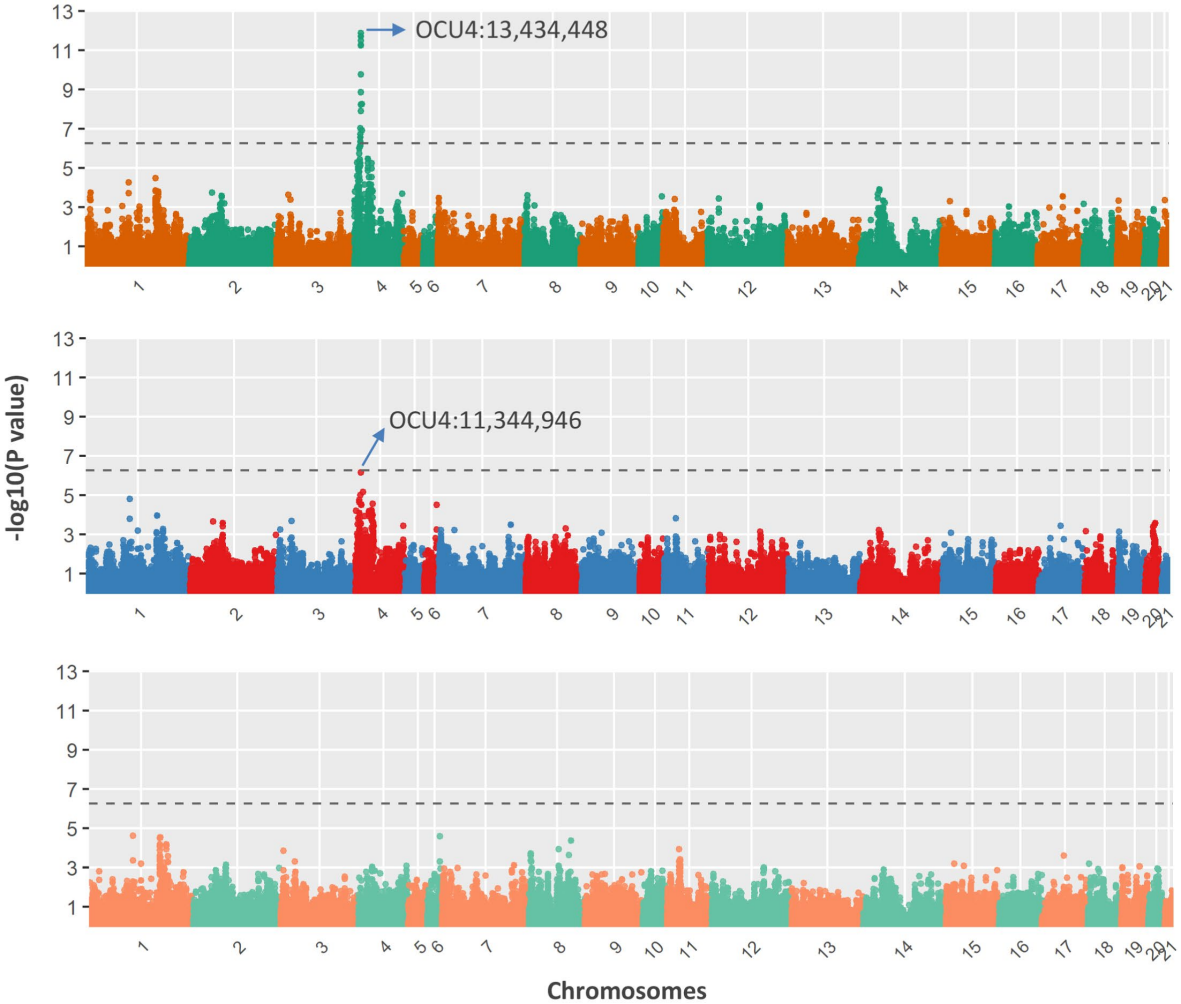
Genome-wide association with coat colors of Chinese Rex rabbits. After testing all SNP effects with a mixed linear model (top panel), the most significant SNP (OCU4:13,434,448) was fitted as a covariate for re-testing of SNP effects (middle panel). Both significant SNPs (OCU4:13,434,448 and OCU4:11,344,946) were simultaneously fitted as covariates for re-testing of SNP effects (bottom panel). The dashed line represents the genome-wide significance threshold.

The association analysis results are shown in Figure 4. A total of 24 SNPs were detected as statistically significant; all of these were located within a 3.01-Mb genomic region on chromosome 4 (OCU4). After fitting the most significant SNP (OCU4:13,434,448; *p* = 1.31e-12) as a covariate, the association signal within this region noticeably decreased, but it still almost reached the threshold for significance (OCU4:11,344,946; *p* = 7.03e-07). The allelic frequencies of the two SNPs within each population are shown in Table 2; notably, OCU4:13,434,448 was completely fixed in the three non-Chinchilla populations. When both SNPs (OCU4:13,434,448 and OCU4:11,344,946) were simultaneously fitted as covariates, there was no longer any significant association signal within this region. Upon reverse-coding of the coat color, the association results did not change noticeably (Supplementary Figure S1).

**Table 2:** Frequencies of reference alleles for the two (near-) significant SNPs.

Coat color variety	OCU4:13,434,448 (T > A)	OCU4:11,344,946 (G > A)
White Rex	1.00	0.64
Californian Rex	1.00	0.98
Black Rex	1.00	0.82
Chinchilla Rex	0.73	0.45
Dark Chinchilla Rex	0.92	0.44
Light Chinchilla Rex	0.55	0.54

We further investigated the annotated genes within this candidate genomic region (including 500 kb upstream of OCU4:11,344,946 and 500 kb downstream of OCU4:13,434,448), identifying 51 positional candidate genes in total. Among these genes, the well-studied *ASIP* gene, which is significantly associated with agouti coat color in rabbits, was located 150 kb upstream of the most significant SNP (OCU4:13,434,448). However, the second-most independently significant SNP (OCU4:11,344,946) was located at a large distance, 1.7 Mb upstream of the *ASIP* gene. Besides *ASIP*, no other known coat-color-associated gene was found within this candidate genomic region.

## Discussion

Coat color is an important phenotypic characteristic in domestic animals and has been directly subjected to artificial selection (41). It has also been proposed that hundreds of loci/genes play a role in affecting coat color, which (in combination with diverse selection preferences among humans) has ultimately resulted in considerable variation in a wide range of domestic animals (9). In addition to being farmed for the production of meat, wool, and fur, modern rabbits have been kept as a pet animal worldwide, with specific emphasis on the subjective selection of coat color. Therefore, rabbits could represent an ideal case for the identification of candidate genes and causal mutations affecting the expression of different coat colors. With the use of a cost-efficient method, genome-wide genetic variants could be discovered *de novo* through implementation high-throughput surveys, such as GWAS, for economically important traits and for the investigation of population genetic structures. In this study, we collected six coat color varieties of Rex rabbits raised in China and employed a high-throughput approach to successfully identify genome-wide and evenly distributed SNPs.

Coat color in mammals is generally considered to be a qualitative trait, although the phenotypic variations are genetically determined by polygenes. Therefore, the genome-wide scanning approach has been increasingly widely used to reveal coat-color-associated candidate genes and causal mutations. For example, Li et al. (42) genotyped ~50 k SNPs and employed a GWAS approach to identify three known pigmentation genes in sheep. In the Iranian Markhoz goat, a total of six genes have been identified as being associated with black, brown, and white coat colors using a GWAS approach (43). Based on the newly discovered SNPs in this study, we also conducted the first GWAS for coat color in Chinese Rex rabbits. Our results revealed that a 2.1-Mb genomic region (OCU4:11,344,946 – 13,434,448) containing *ASIP*, which has been shown in previous studies to be significantly associated with coat color (13), is also significantly associated with coat color in Rex rabbits. In a previous study of Rex rabbits with different coat colors, Yang et al. (44) found that *ASIP* had three alleles and was extensively expressed in all analyzed tissues. Recently, an 11-kb deletion spanning the promoter and first exon of *ASIP* has been suggested to be the most likely causal variant for the black-and-tan phenotype in rabbits (45). In the present study, we confirmed that *ASIP* is a putative causal gene affecting coat color in Chinese Rex rabbits. In the melanocytes of the hair follicle, *ASIP* encodes a paracrine signaling molecule that promotes the synthesis of pheomelanin (46). However, further studies are needed to explore whether the two candidate SNPs identified in this study are causal variants or not; although both of them are located more than 100 kb away from *ASIP* (upstream and downstream), possible roles for these SNPs in regulating gene expression cannot be excluded. Another possibility is that the two candidate SNPs are closely linked to the potential causal variant(s).

In addition to the discovery of coat-color-associated candidate genes, both genetic diversity and population structures among the six coat color varieties of Rex rabbits were investigated using the set of genome-wide SNPs generated in this study. Our results revealed the differential genetic diversity among these coat color varieties, with the highest genetic diversity observed in the Black Rex. This result is consistent with those presented in a previous report on genetic diversity patterns among 29 domestic and wild rabbit populations, examined using microsatellite markers (47). Liu et al. (25) also investigated population structure among eight Chinese rabbit breeds (not including the Rex rabbit), whose F_ST_ values were significantly higher than our estimates in this study; this may suggest that genetic differentiation among different populations of Rex rabbits is relatively low in comparison with other indigenous breeds. In accordance with this possibility, less inter-variety genetic differentiation was observed, with lower Fst values, than in former reports (47, 48). Meanwhile, our clustering analysis revealed that only individuals of the White and Californian Rex rabbit varieties could be clustered together and distinguished from individuals of other varieties. Overall, our results revealed using genome-wide SNP information that there is low genetic differentiation among different coat color varieties of Chinese Rex rabbits.

## Conclusion

In this study, we discovered a genome-wide set of SNPs for Chinese Rex rabbits and used these to perform association analyses for the coat color phenotype. Our results revealed a single genomic region that is significantly associated with Rex coat color, and confirmed that the previously known coat-color-associated gene *ASIP* is a putative causal gene affecting coat color variation in Chinese Rex rabbits. Furthermore, low genetic differentiation was revealed among the six coat color varieties of Rex rabbit studied.

## Data availability statement

The datasets presented in this study can be found in online repositories. The names of the repository/repositories and accession number(s) can be found in the article/Supplementary material.

## Ethics statement

The animal study was approved by the Animal Care and Use Committee of Sichuan Academy of Grassland Sciences (YTS20B03). The study was conducted in accordance with the local legislation and institutional requirements.

## Author contributions

KZ, HL, and S-YC: conceptualization. KZ and GW: formal analysis. LW, BW, XF, NL, ZY, WJ, and XG: resources. KZ: writing—original draft preparation. HL and S-YC: writing—review and editing. All authors contributed to the article and approved the submitted version.

## Funding

This work was financially supported by the Earmarked Fund for the China Agriculture Research System (CARS-43-A-3) and Science and Technology Department of Sichuan Province (2021YFYZ0033 and 2021YFYZ0009).

## Conflict of interest

The authors declare that the research was conducted in the absence of any commercial or financial relationships that could be construed as a potential conflict of interest.

## Publisher’s note

All claims expressed in this article are solely those of the authors and do not necessarily represent those of their affiliated organizations, or those of the publisher, the editors and the reviewers. Any product that may be evaluated in this article, or claim that may be made by its manufacturer, is not guaranteed or endorsed by the publisher.

## Footnotes

^1^ https://www.fao.org/dad-is

^2^ www.arba.net
