# Supplementary material for: A genome-wide association study of coat color in Chinese Rex rabbits
Source: Front Vet Sci. 2023 Aug 16;10:1184764. doi: 10.3389/fvets.2023.1184764 (PMC10467280; doi:10.3389/fvets.2023.1184764)
Supplement: Supplementary file 1 [file Data_Sheet_1.docx]

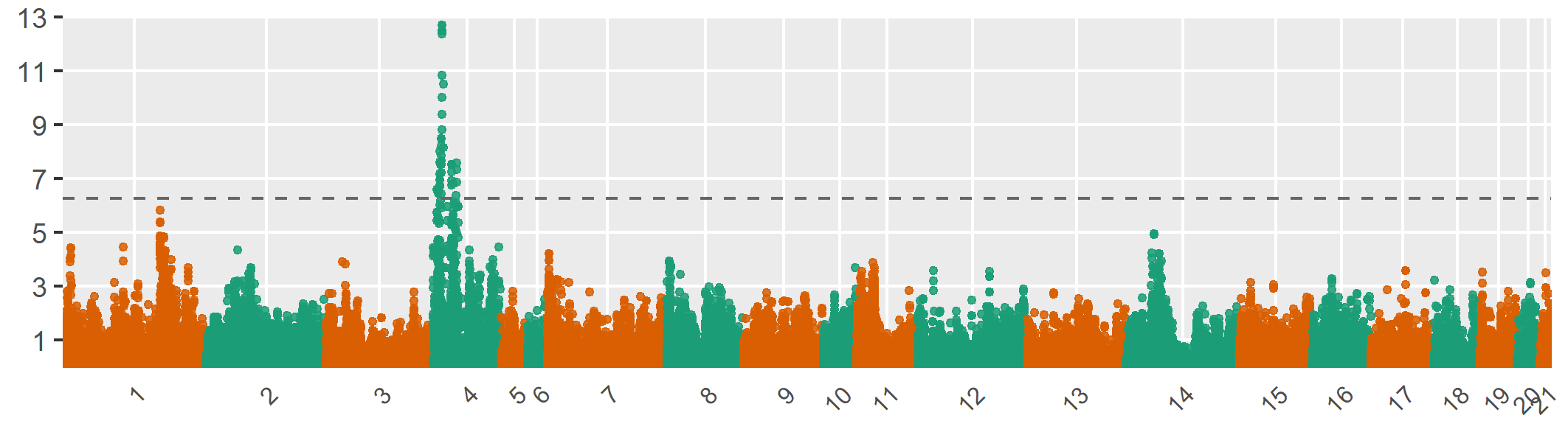


**Supplementary Figure S1. Genome-wide association with coat colours of Chinese Rex rabbits using the reversely coded coat colours**. The dashed line is the genome-wide significance threshold.
